# Immunodominant antibodies against hemagglutinin in ferrets following infection with 2018 to 2019 influenza vaccine strains

**DOI:** 10.1038/s41541-025-01282-y

**Published:** 2025-11-21

**Authors:** Zhu Guo, Thomas Rowe, Jessie Chang, Paul J. Carney, John Steel, James Stevens

**Affiliations:** Influenza Division, National Center for Immunization and Respiratory Diseases, Centers for Disease Control and Prevention, Atlanta, GA USA

**Keywords:** Vaccines, Humoral immunity

## Abstract

Global influenza surveillance depends on antigenic characterization of viral isolates through hemagglutination inhibition and microneutralization assays using sera from panels of ferrets, each infected with a single vaccine virus. However, recent studies have revealed limitations in ferret-based antigenic analyses. Therefore, a deeper understanding of ferret antibody responses following viral infection is essential for better evaluation and interpretation of antigenicity data derived from ferret sera. This study utilized a biolayer interferometry (BLI)-based binding assay and panels of recombinant hemagglutinin (rHA1), each carrying multiple substitutions within one of the antigenic sites of H1 or H3 HA, to analyze the immunodominant antibodies against HA in ferret sera following infection with the 2018-2019 Northern Hemisphere influenza vaccine virus A/Michigan/45/2015(H1N1)pdm09 (MI/45) or A/Singapore/INFIMH-16-0019/2016(H3N2) (SING/16). The results demonstrate dominant antibody responses against HA in ferrets and provide valuable insights into interpreting ferret-derived antigenicity data and improving influenza vaccine virus selection.

## Introduction

Seasonal influenza virus infections cause significant morbidity and considerable mortality worldwide each year^1^. Vaccination remains the primary measure to prevent influenza^2^. However, due to the selection pressures exerted by immune responses to vaccination or natural infection, influenza viruses constantly accumulate mutations to evade human immunity. Consequently, the composition of seasonal influenza vaccines requires frequent updates to ensure that the antigenicity of vaccine viruses closely matches that of the predominant circulating viruses^3^. To support these updates, a global network of laboratories, the Global Influenza Surveillance and Response system (GISRS), launched in 1952, monitors the genetic and antigenic evolution of influenza viruses and provides critical data for seasonal influenza vaccine strain selection^4^. Surveillance of virus antigenicity is primarily conducted through hemagglutination inhibition (HI) and microneutralization (MN) assays using sera from human or animal and virus isolates. These assays help to analyze the hemagglutinin (HA), the immunodominant surface protein of influenza virus. This large trimeric protein is composed of a head domain (HA1) that contains the receptor binding site (RBS), and a stalk domain (HA2). The HI assay determines the titers of neutralizing Abs targeting mainly the HA1, in and around the RBS^5^. In contrast, the MN assay measures the titers of neutralizing Abs against both the HA1 and HA2 domains^6^. Circulating viruses that show at least a fourfold reduction in HI or MN titers compared to vaccine virus strains are considered antigenically drifted and selected as possible candidates for vaccine reformulation^7,8^.

Seasonal influenza vaccines primarily induce strain-specific Ab responses targeting the HA1 domain of HA^9^. The binding of Abs to hypervariable epitopes located in or near the RBS of HA1 blocks viral access to cellular receptors, thereby neutralizing the virus^10^. Classically defined antigenic sites on HA1 have been identified by tracking amino acid changes in natural variants or through in vitro selection. For the H1 subtype of influenza A virus (IAV), five antigenic sites (Sa, Sb, Ca1, Ca2 and Cb) have been mapped, while for the H3 subtype, five sites (A through E) have been defined^11–13^. These sites contain multiple overlapping epitopes and exhibit high variability among viral strains. The accumulation of amino acid substitutions in these regions can lead to significant antigenic changes^10,12,14^. Remarkably, even a single amino acid substitution in the HA head can cause antigenic drift^15–18^. Furthermore, a single amino acid change in HA1 has been shown to substantially disrupt serum Ab binding in focused Ab responses elicited by influenza virus infection or vaccination^19,20^. The substitutions are mainly elicited by immune selection pressure but can also arise during the propagation of viruses such as in chicken eggs^21–23^. In fact, egg-adaptive mutations in vaccine virus HAs have been shown to be associated with low vaccine effectiveness with recent seasonal influenza vaccines^22,24,25^.

Ferrets are natural hosts for type A and B influenza viruses and are valuable models for influenza research^26,27^. They are widely used to study influenza pathogenesis and transmission due to similarities between ferrets and humans in the distribution of sialic acid receptors in the respiratory tract and the clinical signs and symptoms observed during infection^28–31^. Additionally, ferret immune responses to influenza virus infection or vaccination are considered to closely mimic those in humans, making them critical in the annual seasonal influenza vaccine strain selection and vaccine development process^32–34^. Specifically, sera from panels of ferrets, each infected with a single vaccine virus, are routinely used in the HI or MN assays conducted during the annual GISRS influenza surveillance. Despite their utility, recent studies have revealed limitations in ferret-based antigenic analyses^16,35^. For example, a K163Q substitution in the HA of an A(H1N1)pdm09 virus caused significant antigenic changes that were detectable by human sera but not by ferret sera^16^. Furthermore, sequential infection of ferrets with a seasonal A(H1N1) virus and an A(H1N1)pdm09 virus shifted the Ab response to be similar to those in humans^35^. These findings suggest that ferret sera from primary influenza virus infections may not fully represent human humoral response to viral infection or vaccination, and ferret sera-based antigenic analysis may not always identify drifted strains which are clinically relevant. Therefore, further characterization of ferret Ab responses following virus infection is essential for better evaluation and interpretation of antigenicity data derived from ferret sera.

A dominant Ab response to HA protein is closely linked to antigenic drift of influenza virus^36,37^, and identification of dominant epitopes is critical for vaccine virus selection. In this study, we assess the immunodominant Abs against influenza HA in ferret sera following infection with the 2018–2019 influenza A vaccine strains using a biolayer interferometry (BLI)-based binding assay, the flu antibody biosensor assay-2 (f-AbBA-2)^19,20^. By analyzing ferret sera binding to panels of rHA1 proteins, each containing multiple substitutions in one of the antigenic sites of H1 or H3 HA from the vaccine strains, we identified the dominant HA antigenic sites of an A(H1N1)pdm09 virus, A/Michigan/45/2015 (MI/45) and an A(H3N2) virus, A/Singapore/INFIMH-16-0019/2016 (SING/16) in ferrets. Individual residues critical for the dominant antigenic site binding were also determined using panels of rHA1 proteins, each carrying one amino acid substitution in the HA. Furthermore, we analyzed contributions of individual residues to the enhanced SING/16 HA1 binding to ferret sera post infection with the egg-grown SING/16 virus. These results provide valuable insights into interpreting antigenicity data derived from ferret sera and improving the influenza vaccine virus selection process.

## Results

### Expression and characterization of MI/45 and SING/16 rHA1s with mutated antigenic sites

**Fig. 1:**
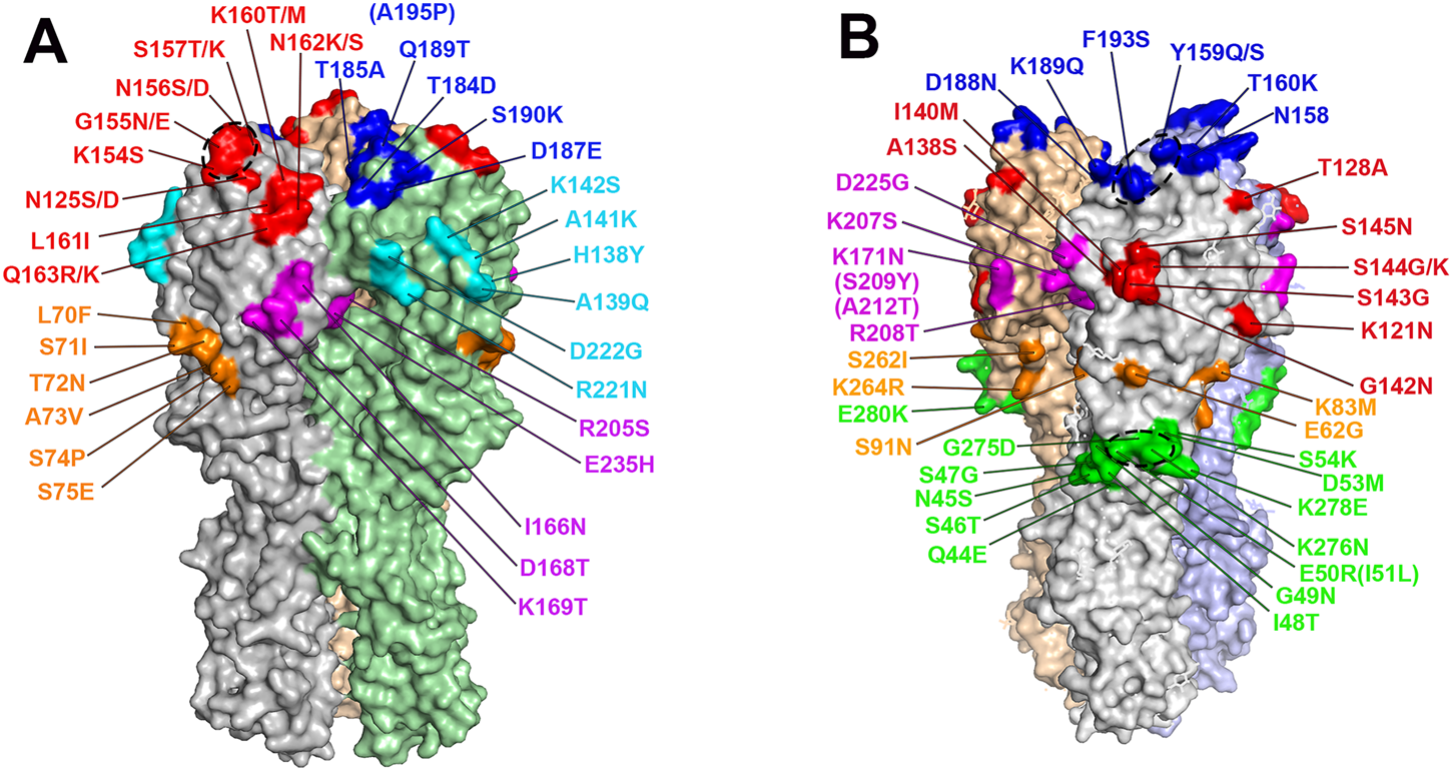
Substitutions in the rHA1 mutants of MI/45 and SING/16 used in this study. **A** The sequences and locations of MI/45 substitutions are shown on the 3D structure of trimeric H1 HA (PDB ID: 7KNA) with colors indicating corresponding antigenic sites: Sa (red), Sb (blue), Ca1 (magenta), Ca2 (cyan), and Cb (orange). **B** The substitutions in SING/16 HA are shown on the 3D structure of trimeric H3 HA (PDB ID: 4WE8) with colors indicating corresponding antigenic sites: A (red), B (blue), C (green), D (magenta), and E (orange). Potential epitopes are indicated by dashed ovals.

Previous studies have mapped immunodominant antigenic sites of H1 and H3 HAs recognized by human and animal HI-active Abs using panels of influenza viruses each carrying mutations within one classically defined antigenic site of HA^38,39^. To further investigate the binding patterns of total ferret serum Abs against HA1, a panel of five antigenic site mutants of MI/45 rHA1 (ΔSa, ΔSb, ΔCa1, ΔCa2, and ΔCb) was generated, each containing multiple substitutions within one antigenic site as described previously (Fig. 1A and Table 1)^38^. Similarly, a panel of five SING/16 rHA1 mutants (ΔA, ΔB, ΔC, ΔD, ΔE) was created each carrying mutations within one antigenic site of SING/16 HA as described previously with modifications (Fig. 1B and Table 1)^39^. Additionally, two mutant rHA1s, Δ5-H1 and Δ5-H3, incorporating all mutations present in the single antigenic site mutants of H1 or H3, were developed for MI/45 and SING/16, respectively (Table 1). To verify proper folding of the expressed MI/45 rHA1s, the f-AbBA-2 was performed using the MI/45 rHA1 panel and an anti-MI/45 HA1 mAb, IT-3A10. Similarly, structure integrity of the SING/16 rHA1s was assessed using the f-AbBA-2 with a pan-H3 anti-HA1 mAb, FluA-20 (Supplementary Fig. 1A, B)^40^. Equivalent amounts of the WT and mutant rHA1s used in the assays were confirmed by Western blot analysis (Supplementary Fig. 1A, B). The binding assays showed that all the expressed H1 and H3 rHA1s with antigenic site mutations were recognized by IT-3A10 or FluA-20 at levels comparable to their respective WT rHA1s, indicating proper folding of these recombinant proteins.

**Table 1:** Summary of mutations in antigenic site mutants of MI/45 and SING/16 rHA1

MI/45	Mutations	SING/16	Mutations
ΔSa	N125S, G155N, N156S, S157T, K160T, L161I, N162K, Q163R	ΔA	I140M, G142N, S143G, S144G, S145N
ΔSb	T184D, T185A, D187E, Q189T, S190K, A195P	ΔB	Y159Q, D188N, K189Q
ΔCa1	I166N, D168T, K169T, R205S, E235H	ΔC	Q44E, N45S, S46T, S47G, I48T, G49N, E50R, I51L, D53M, S54K, G275D, K276N, K278E, E280K
ΔCa2	H138Y, A139Q, A141K, K142S, R221N, D222G	ΔD	K207S, R208T, S209Y, A212T
ΔCb	L70F, S71I, T72N, A73V, S74P, S75E	ΔE	E62G, K83M, S262I, K264R
Δ5-H1	Mutations in ∆Sa/ΔSb/ΔCa1/ΔCa2/ΔCb	Δ5-H3	Mutations in ∆A/ΔB/ΔC/ΔD/ΔE

### Identification of immunodominant antigenic sites of MI/45 and SING/16 HA1 targeted by ferret post-infection sera

**Fig. 2:**
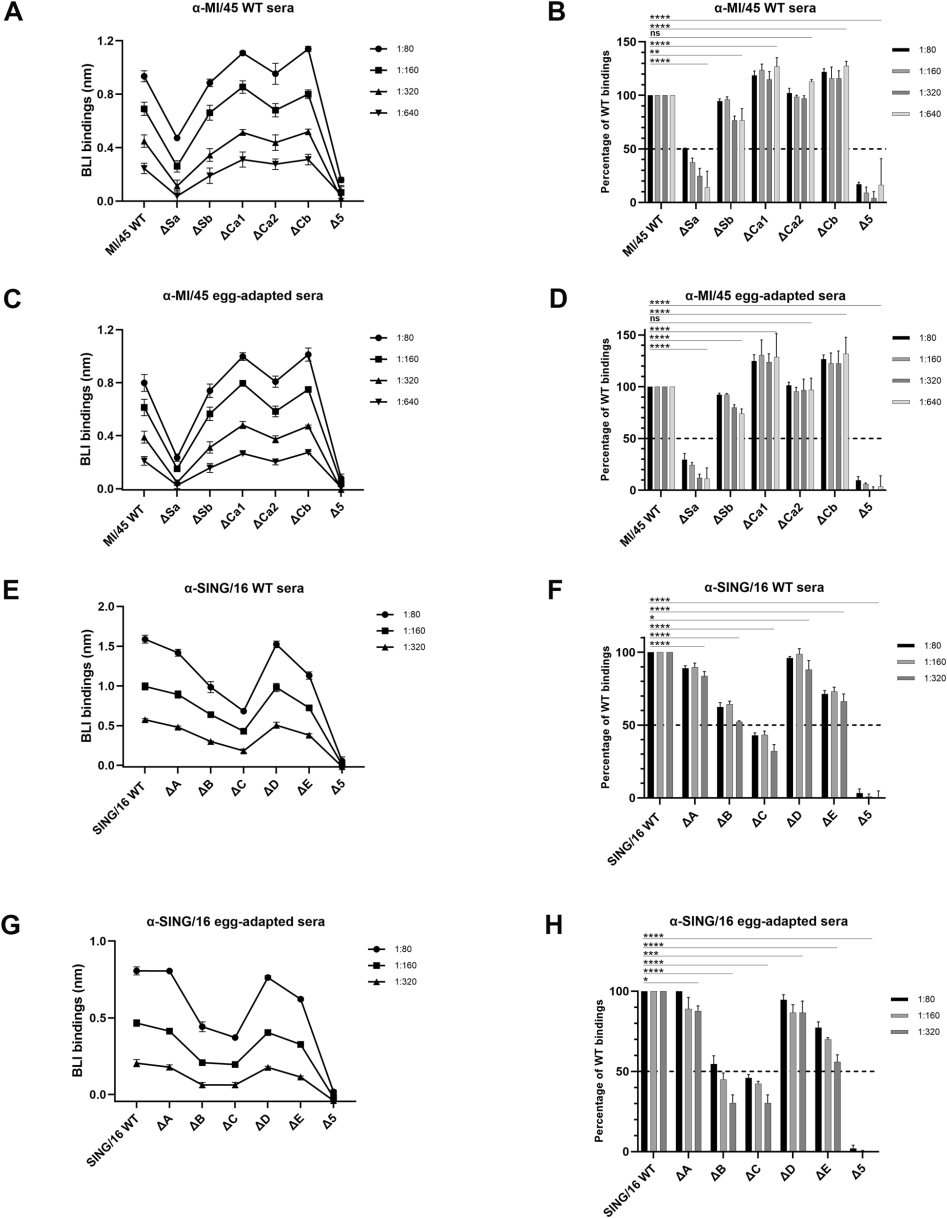
Identification of immunodominant antigenic sites of MI/45 and SING/16 HA1 targeted by ferret post-infection sera. The dominant antigenic sites of MI/45 HA1 for sera from ferrets infected with cell-grown (WT sera) (**A**, **B**) or egg-grown (egg-adapted sera) (**C**, **D**) MI/45 viruses were determined using the f-AbBA-2 with a panel of six MI/45 rHA1 mutants. Similarly, the dominant antigenic sites of SING/16 HA1 for the WT (**E**, **F**) or egg-adapted (**G**, **H**) anti-SING/16 ferret sera were identified using a panel of six SING/16 rHA1 mutants. Sera were tested at dilutions of 1:80 to 1:640 for MI/45 (**A**–**D**) and 1:80 to 1:320 for SING/16 (**E**–**H**). Each symbol represents the median BLI binding with standard deviation from three independent experiments (**A**, **C**, **E**, **G**). Binding results of each rHA1 mutant were normalized against WT controls, with median and standard deviation shown (**B**, **D**, **F**, **H**). Statistical significance: ^*^p < 0.05, ^**^p < 0.01, ^***^p < 0.001, ^****^p < 0.0001, ns (not significant).

To characterize Ab responses to MI/45 virus infection in ferrets, the binding of WT and antigenic site mutants of MI/45 rHA1s was tested against sera from ferrets infected with MI/45 viruses propagated in cell culture (hereafter referred to as WT sera), and sera from ferrets infected with viruses propagated in chicken eggs (referred to as egg-adapted sera) (Fig. 2A–D). The egg-grown viruses carried a Q223R substitution in HA. Previous studies have demonstrated that egg-grown viruses can elicit Ab responses distinct from those induced by their cell-grown counterparts, which are generally regarded as the WT, in both humans and animals^21,22^. The MI/45 WT rHA1 showed comparable binding to the WT and egg-adapted ferret sera, and this binding consistency was observed across sera dilutions ranging from 1:80 to 1:640. The ΔSa mutant, which carries substitutions within the antigenic site Sa, exhibited a marked reduction in binding (>50%) to both sera compared to the WT. In contrast, the ΔSb and ΔCa2 showed binding levels comparable to the WT. Interestingly, the ΔCa1 and ΔCb exhibited slightly higher binding levels (~20 to 30%) than the WT. Strikingly, the Δ5-H1 mutant of MI/45 showed minimal interactions with both sera. The results indicate that the cell-grown and egg-grown MI/45 viruses elicited similar Ab responses in ferrets. The induced ferret Abs to the HA head domain primarily targeted the five H1 antigenic sites, with site Sa as the most dominant in response to MI/45 infection.

The Ab responses to SING/16 virus infection were also analyzed using the f-AbBA-2 with the SING/16 antigenic site mutant panel and sera from ferrets infected with cell-grown or egg-grown SING/16 viruses (Fig. 2E–H). The egg-grown viruses carried three egg-adaptive substitutions in HA (T160K, L194P, and D225G). Unlike MI/45, the SING/16 WT rHA1 and single antigenic site mutants showed binding to the WT sera at levels approximately 2-fold of their corresponding binding levels against the egg-adapted sera. This difference was consistent across sera dilutions ranging from 1:80 to 1:320. At a 1:80 sera dilution, the ΔC, ΔB, and ΔE showed significantly reduced binding to both sera, averaging 44.5%, 58.5% and 74.3% of the WT rHA1 binding level, respectively. In contrast, the ΔA and ΔD exhibited binding levels comparable to the WT rHA1. As observed with MI/45 Δ5-H1 rHA1, the Δ5-H3 mutant of SING/16 displayed minimal binding activity with both sera. These findings suggest that the SING/16 egg-grown viruses induced lower avidity Abs against the SING/16 WT and single antigenic site mutant rHA1s compared to the cell-grown viruses, although the binding hierarchies were highly similar between the two sera. The anti-HA1 Abs elicited by SING/16 virus infection predominantly targeted the five H3 antigenic sites. In comparison to MI/45, the Ab responses to SING/16 virus infection displayed a more complex dominance hierarchy, with site C being the most dominant, followed by site B and then site E.

### Identification of individual amino acid residues critical in the binding of immunodominant antigenic sites of MI/45 and SING/16 HA1

**Fig. 3:**
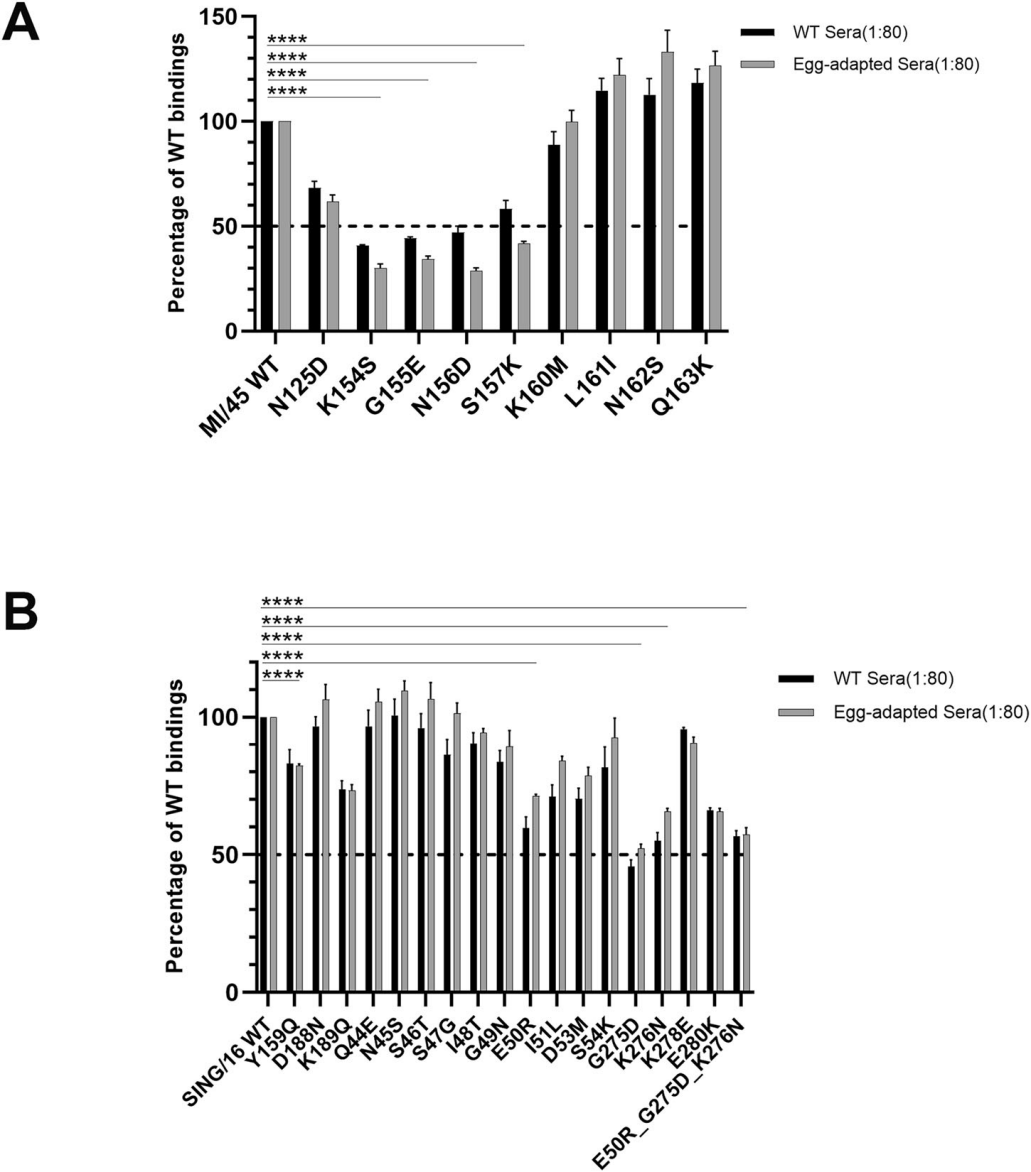
Identification of key amino acid residues in immunodominant antigenic sites of MI/45 and SING/16 HA1. **A** Targeted residues in antigenic site Sa of MI/45 were identified using a panel of nine MI/45 rHA1 mutants with ferret WT or egg-adapted anti-MI/45 sera. **B** Targeted residues in antigenic site B and C of SING/16 were identified using a panel of eighteen SING/16 rHA1 mutants with ferret WT or egg-adapted anti-SING/16 sera. Sera were tested at 1:80 dilution. Binding results were normalized against WT controls, with median and standard deviation shown. Statistical significance: ^****^p < 0.0001.

To investigate the role of individual amino acid residues in the dominant binding of MI/45 site Sa, a panel of nine MI/45 rHA1s was generated each containing one single amino acid substitution within the site Sa. Most substitutions were derived from mutations in the ΔSa mutant (Table 2). Proper folding of the mutant rHA1s was verified by the f-AbBA-2, which showed comparable binding to the IT-3A10 mAb between the WT and mutant proteins of equivalent amounts (Supplementary Fig. 2A, C). This rHA1 mutant panel was subsequently applied in the f-AbBA-2 to assess their binding avidities to the 1:80 diluted WT and egg-adapted anti-MI/45 sera (Fig. 3A). The binding assays revealed that four site Sa mutants (K154S, G155E, N156D, and S157K) showed significantly reduced binding levels of less than 50% of the WT. One mutant (N125D) displayed an intermediate reduction, with binding levels of approximately 65% of the WT. The remaining four mutants exhibited binding levels that were either comparable (K160M) to or moderately higher (~10-30%) (L161I, N162S, and Q163K) than the WT. These results confirm the site Sa immunodominance and demonstrate the distinct contributions of individual amino acid residues to the site Sa binding, with residues 154 through 157 identified as an epitope critical for the ferret anti-MI/45 sera binding (Fig. 1A).

**Table 2:** Single mutations in rHA1 panels of MI/45 and SING/16

MI/45 mutation	H1 antigenic site	SING/16 mutation	H3 antigenic site	SING/16 mutation	H3 antigenic site
N125D	Sa	Q44E	C	S54K	C
K154S	Sa	N45S	C	Y159Q	B
G155E	Sa	S46T	C	T160K	B
N156D	Sa	S47G	C	D188N	B
S157K	Sa	I48T	C	K189Q	B
K160M	Sa	G49N	C	D225G	D
L161I	Sa	E50R	C	G275D	C
N162S	Sa	I51L	C	K276N	C
Q163K	Sa	D53M	C	K278E	C
				E280K	C

The role of individual amino acid residues in the dominant binding of site B and site C of SING/16 HA was also examined. A panel of SING/16 rHA1s was generated, including three mutants with single substitutions within the site B, fourteen mutants with single substitutions and one triple mutant containing three substitutions within the site C (Table 2). All substitutions were based on mutations in the ΔB and ΔC mutants. Proper folding of the SING/16 rHA1 mutants was confirmed by the f-AbBA-2, which showed comparable binding to the FluA-20 mAb between the WT and mutant proteins of equivalent amounts (Supplementary Fig. 2B, D). The binding avidities of these mutants were then assessed using the f-AbBA-2 with 1:80 diluted WT and egg-adapted anti-SING/16 sera (Fig. 3B). For the site B mutants, the Y159Q and K189Q substitutions resulted in binding levels of approximately 70–80% of the WT rHA1, while the D188N mutant exhibited binding comparable to the WT. In contrast, several of the site C mutants displayed more pronounced reductions in binding. Substitutions E50R, G275D and K276N reduced binding levels to approximately 50–60% of the WT rHA1. Interestingly, the E50R_G275D_K276N triple mutant, incorporating all three substitutions, did not show further reductions in binding compared to the individual single-substitution mutants, suggesting that the effects of these residues may be non-additive. Binding levels of the remaining site C mutants were either comparable to the WT (Q44E, N45S, S46T, S47G, I48T, G49N, S54K, and K278E) or moderately reduced to approximately 60-80% of the WT rHA1 (I51L, D53M, and E280K). Overall, these findings confirm the immunodominance of site B and C, and indicate that multiple residues contributed to the site B binding, while the site C binding was primarily focused on an epitope centered around residues E50, G275, and K276 of SING/16 HA (Fig. 1B).

### Determination of the role of egg-adaptive substitutions in the SING/16 HA1 binding

**Fig. 4:**
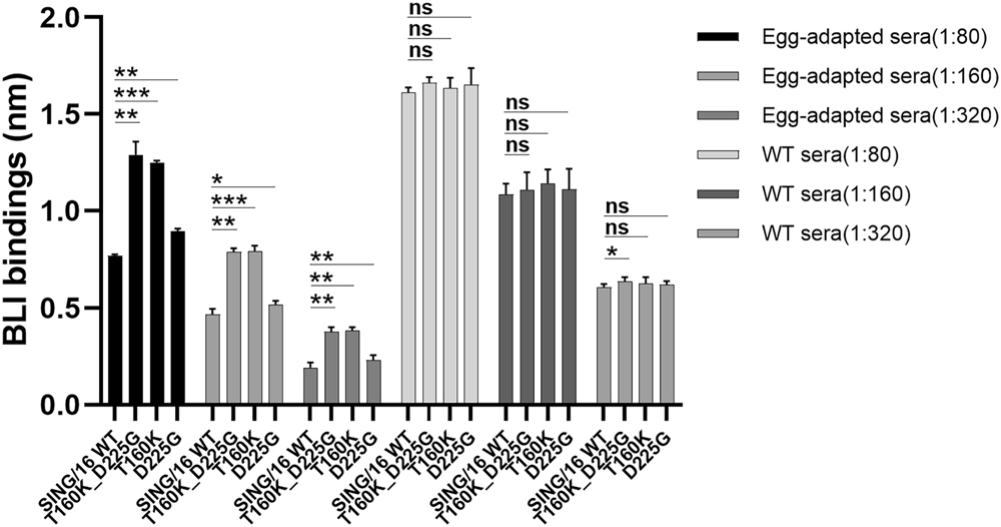
Impact of egg-adaptive substitutions on SING/16 HA1 binding to ferret serum Abs. The rHA1 mutants carrying egg-adaptive substitutions from SING/16 were tested against WT and egg-adapted anti-SING/16 sera using the f-AbBA-2. Sera were tested at dilutions of 1:80 to 1:320. Each bar represents the median BLI binding with standard deviation from three independent experiments. Statistical significance: ^*^p < 0.05, ^**^p < 0.01, ^***^p < 0.001, ns (not significant).

Previous studies have demonstrated that infection of ferrets with egg-grown viruses or vaccination of humans with egg-based influenza vaccines elicits higher HI titers against the egg-grown viruses compared to their cell-grown counterparts^22^. One such substitution, T160K, causes de-glycosylation at residue N158, leading to reduced vaccine effectiveness against A(H3N2) viruses during the 2016-2019 influenza seasons^22,24,41,42^. Three egg-adaptive substitutions have been identified in SING/16 HA: T160K, L194P and D225G. To examine their contribution to ferret sera binding, a panel of rHA1 mutants containing single or double egg-adaptive substitutions was generated (Table 2). The L194P substitution resulted in poor rHA1 protein expression and was excluded from further analysis. Equivalent amounts of the rHA1 proteins were tested against the WT and egg-adapted anti-SING/16 sera using the f-AbBA-2 (Fig. 4 and Supplementary Fig. 3). All the WT and mutant rHA1s showed comparable binding to the WT sera, while the T160K single and T160K_D225G double mutants exhibited significantly higher binding (>60%) to the egg-adapted sera compared to the WT rHA1. In contrast, the D225G single mutant showed only a minimal increase in binding to the egg-adapted sera relative to the WT rHA1. The results were consistent across sera dilutions ranging from 1:80 to 1:320. These findings demonstrate that the egg-adapted serum Abs were detectable using mutants carrying the T160K substitution, which played a more prominent role in mediating antigenic drift compared to D225G.

### Identification of residues responsible for antigenic drift in A/Kansas/14/2017 HA1

**Fig. 5:**
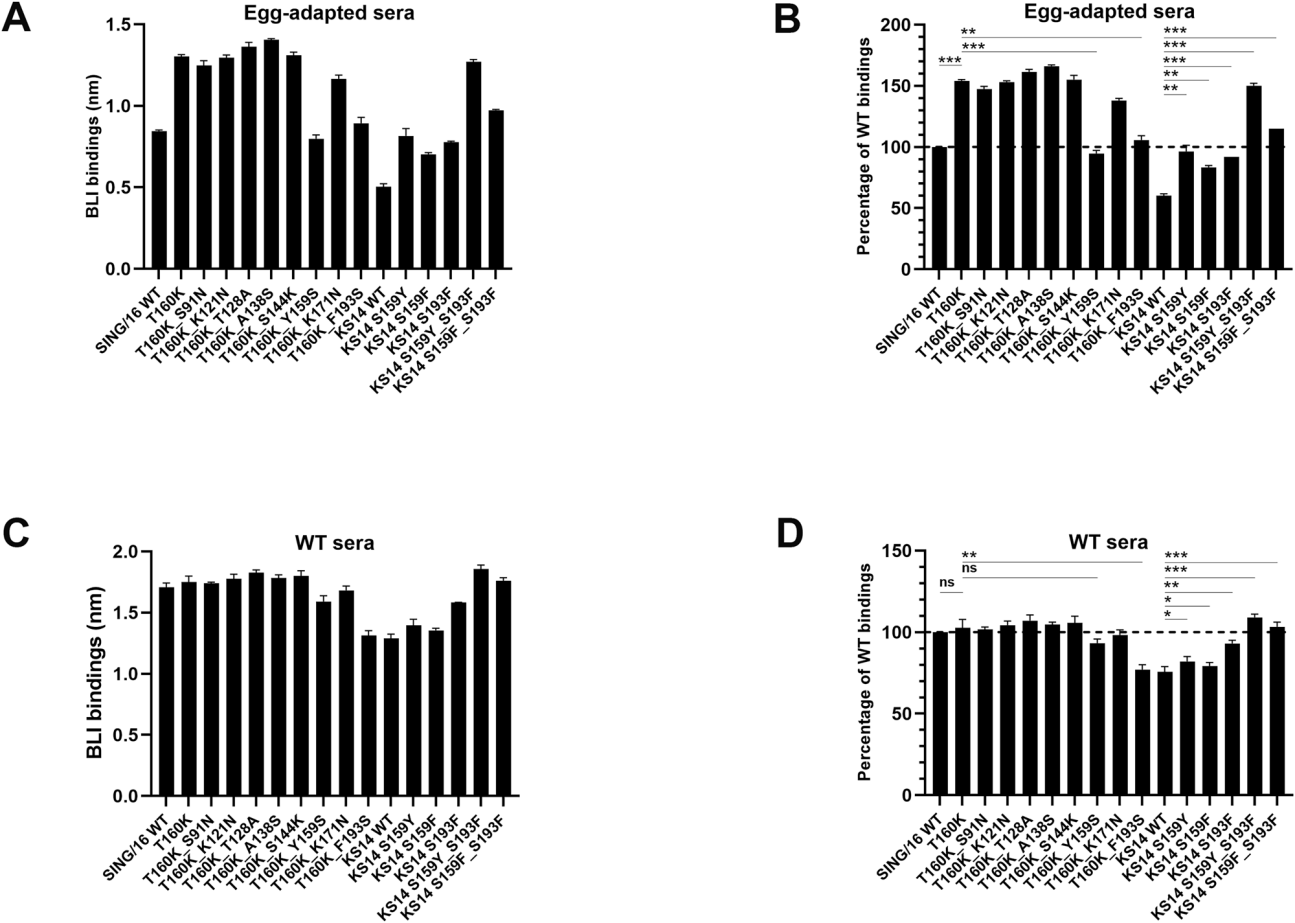
Identification of residues responsible for antigenic drift in KS/14 HA1. **A**, **B** Epitope mapping for egg-adapted anti-SING/16 sera (1:80 dilution) using a panel of SING/16 and KS/14 rHA1 mutants. **C**, **D** Epitope mapping for WT anti-SING/16 sera (1:80 dilution). Each bar represents the median BLI binding with standard deviation from three independent experiments (**A**, **C**). Binding results were normalized against SING/16 WT controls, with median and standard deviation shown (**B**, **D**). Statistical significance: ^*^p < 0.05, ^**^p < 0.01, ^***^p < 0.001, ns (not significant).

The A(H3N2) vaccine virus strain for the 2019-2020 Northern Hemisphere influenza season was updated to A/Kansas/14/2017 (KS/14). Sequence analysis of KS/14 HA1 revealed nine amino acid changes, including a T160K substitution, compared to SING/16 HA1 (Supplementary Fig. 4A). To assess whether these changes contributed to antigenic drift, the binding avidities of KS/14 and SING/16 rHA1s to the egg-adapted anti-SING/16 sera were compared using the f-AbBA-2 (Fig. 5A, B). The binding assays showed significantly reduced binding of KS/14 rHA1 to the sera (1:80 dilution), with levels of approximately 60% and 39% of the SING/16 WT and T160K mutant, respectively. These results suggest that the substitutions in KS/14 HA1 altered its antigenicity, a drift also detected by the HI assay^24^, and that the attenuation of KS/14 HA1 binding to the egg-adapted sera was primarily driven by the eight substitutions other than T160K. To further investigate the role of individual KS/14 HA1 substitutions in binding, a panel of SING/16 rHA1 double mutants was generated, each containing the T160K substitution along with one of the eight substitutions from KS/14 HA1 (Table 3). Proper folding of the recombinant proteins was confirmed using the f-AbBA-2 with FluA-20 mAb and equivalent amounts of the rHA1s (Supplementary Fig. 4B, C). The double mutant panel was subsequently tested for binding to the egg-adapted sera (Fig. 5A, B). The T160K_Y159S and T160K_F193S double mutants exhibited significantly reduced binding with levels of 61% and 69% of the T160K single mutant, respectively. In contrast, minimal changes in binding were observed for the other six double mutants (T160K_S91N, T160K_K121N, T160K_T128A, T160K_A138S, T160K_S144K, and T160K_K171N). These results indicate that Y159 and F193 were the key residues in the binding of egg-adapted sera.

**Table 3:** Mutations of SING/16 and KS/14 used in the epitope mapping of SING/16 HA1

SING/16 mutation	KS/14 mutation
T160K	S159Y
T160K-S91N	S159F
T160K-K121N	S193F
T160K-T128A	S159Y-S193F
T160K-A138S	S159F-S193F
T160K-S144K	
T160K-Y159S	
T160K-K171N	
T160K-F193S	

To validate the dominant roles of Y159 and F193 in the egg-adapted sera binding, a panel of KS/14 rHA1 mutants was generated, each carrying single or double reversions at position 159 or 193 (Table 3). Proper folding of the recombinant rHA1s was confirmed using the f-AbBA-2 with FluA-20 mAb and equivalent amounts of recombinant proteins (Supplementary Fig. 4B, C). The binding assays showed significantly enhanced binding of the S159Y and S193F single revertant with levels of 162% and 154% of the KS/14 WT rHA1, respectively (Fig. 5A, B). Notably, the S159Y_S193F double revertant binding was markedly increased to a level comparable to that of the SING/16 T160K mutant. In addition to tyrosine, phenylalanine is another residue commonly found at position 159 of the HAs from human A(H3N2) viruses^43^. An F159S substitution in the HA of 2014-2015 Northern Hemisphere seasonal vaccine strain A/Texas/50/2012(H3N2) caused an antigenic change, highlighting the critical role of F159 in the Ab response^44^. To determine whether F159 and Y159 are conserved in Ab responses targeting residue 159 of H3, S159F single and S159F_S193F double revertants of KS/14 rHA1 were generated and tested against egg-adapted anti-SING/16 sera. The binding assays showed that reverting serine at position 159 to phenylalanine in both the S159F and S159F_S193F revertants improved KS/14 rHA1 binding, although to a lesser extent than their respective revertants containing the S159Y reversion, indicating conservation of phenylalanine or tyrosine at this site. These findings support the critical role of Y159 and F193 in the egg-adapted sera binding, and mutations of the two site B residues largely contributed to the drifted response of KS/14 HA1 against the egg-adapted anti-SING/16 sera. (Fig. 1B).

Furthermore, to identify residues involved in the drifted response of KS/14 HA1 against the WT anti-SING/16 sera, the rHA1s from SING/16 and KS/14 were tested against the WT sera using the f-AbBA-2 (Fig. 5C, D). The KS/14 WT rHA1 showed binding levels of 76% of the SING/16 WT rHA1, while the SING/16 WT and T160K single mutant exhibited comparable binding. For the SING/16 double mutants, the T160K_Y159S rHA1 showed only a minimal reduction in binding compared to the SING/16 WT rHA1. In contrast, similar to KS/14 WT rHA1, the T160K_F193S rHA1 displayed reduced binding levels of 77% of the SING/16 WT rHA1. Minimal changes in binding were observed for the other six double mutants relative to the SING/16 WT rHA1. Among the KS/14 revertants, the S159Y and S159F rHA1 exhibited minimally increased binding compared to the KS/14 WT, while the S193F rHA1 showed significantly enhanced binding levels of ~93% of the SING/16 WT. Interestingly, the S159Y_S193F and S159F_S193F double revertants displayed enhanced binding levels slightly higher (~11% and 3%, respectively) than those of the SING/16 WT. These findings indicate that the F193S substitution mainly contributed to the KS/14 HA1 antigenic drift relevant to the WT sera.

### Analysis of antigenic drift elicited by infection of ferrets with egg-grown A(H3N2) vaccine viruses of recent influenza seasons

**Fig. 6:**
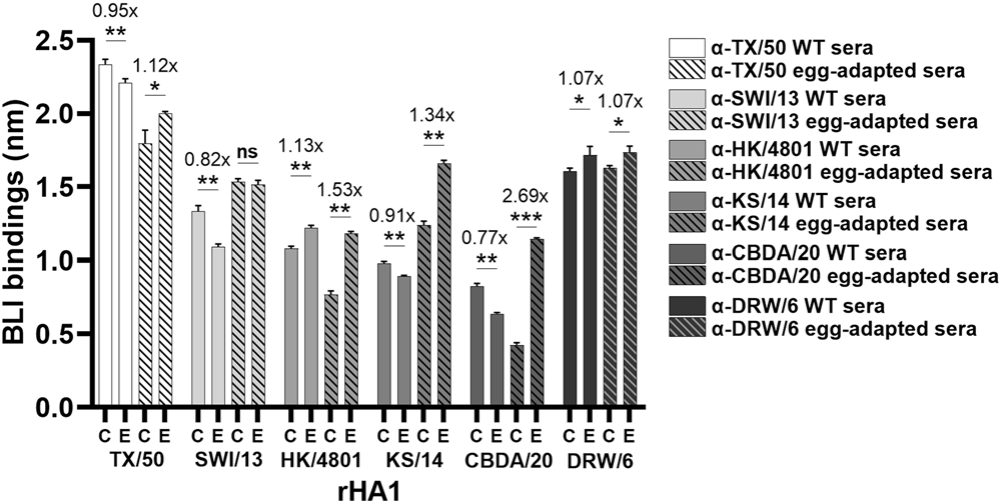
Antigenic drift induced by egg-grown A(H3N2) vaccine viruses in ferrets. Cell-version (C) and egg-version (E) rHA1s from six A(H3N2) vaccine viruses were tested against WT and egg-adapted anti-H3N2 sera (1:80 dilution) using the f-AbBA-2. Each bar represents the median BLI binding with standard deviation from three independent experiments. The number above the bars of each rHA1 pair represents the ratio of egg-version to cell-version rHA1 binding. Statistical significance: ^*^p < 0.05, ^**^p < 0.01, ^***^p < 0.001, ns (not significant).

To determine whether other egg-grown A(H3N2) vaccine viruses from recent influenza seasons also induce antigenic drift in ferrets, a panel of rHA1s was generated. The panel included six pairs of rHA1s derived from A(H3N2) vaccine viruses selected for the 2014–2023 Northern Hemisphere influenza seasons. Each pair consisted of a cell-version and an egg-version rHA1, originating from cell-grown and egg-grown viruses, respectively (Table 4). The L194P substitution caused poor HK/4801 rHA1 expression and was not incorporated into the construct. Proper folding of the recombinant proteins was confirmed using the f-AbBA-2 with the mAb FluA-20 and equivalent amounts of each rHA1 pair (Supplementary Fig. 5A, B). The binding avidities of each rHA1 pair to their respective ferret sera were then assessed using the f-AbBA-2 (Fig. 6). The binding assays showed that several rHA1 pairs, including those from TX/50, SWI/13 and DRW/6, exhibited similar binding to both WT and egg-adapted sera. In contrast, the egg-version rHA1s from HK/4801, KS/14 and CBDA/20 showed significantly increased binding to egg-adapted sera, reaching 153%, 134% and 269% of the binding levels of their corresponding cell-version rHA1s, respectively. However, these egg-version rHA1s exhibited binding to WT sera at levels comparable to or slightly lower than their cell-version counterparts. These results suggest that while not all egg-adapted A(H3N2) viruses induced antigenic drift in ferrets, the egg-adapted substitutions in HK/4801, KS/14 and CBDA/20 did result in antigenic drift.

**Table 4:** Ferret sera used in the study

Sample ID^a^	NH^d^ winter season	Lot or item number	Supplier	Egg-adaptive^b^ mutations
fs-MI/45-c fs-MI/45-e	2017–2019	2017–093 2017–092	CDC CDC	Q223R
fs-TX/50-c fs-TX/50-e	2014–2015	2014–085 2013–046	CDC CDC	G186V, S219F, I226N
fs-SWI/13-c fs-SWI/13-e	2015–2016	2018–009 2017–008	CDC CDC	I140R, G186V, S219Y
fs-HK/4801-c fs-HK/4801-e	2016–2018	2016–089 2017–112	CDC CDC	N96S, T160K, L194P^c^
fs-SING/16-c fs-SING/16-e	2018–2019	2018–021 FR-1618	CDC IRR	T160K, L194P^c^, D225G
fs-KS/14-c fs-KS/14-e	2019–2020	2020–010 2018–098	CDC CDC	D190N, N246T
fs-CBDA/20-c fs-CBDA/20-e	2021–2022	2022–066 2021–058	CDC CDC	T160K, S186R
fs-DRW/6-c fs-DRW/6-e	2022–2023	2023–060 2022–005	CDC CDC	S219F, D225G

^a^fs indicates ferret sera; c and e indicate sera from ferrets infected with cell- or egg-grown viruses, respectively.

^b^Mutations that are carried in the rHA1s and HAs of corresponding egg-grown viruses.

^c^L194P caused poor rHA1 expression and was not incorporated into the rHA1 mutant.

^d^NH (Northern Hemisphere).

## Discussion

We have identified the immunodominant antigenic sites of MI/45 and SING/16 HA1 targeted by ferret post-influenza virus infection sera through direct antigen-antibody binding analysis. Our results show that ferret anti-MI/45 sera predominantly recognize the H1 antigenic site Sa, suggesting their suboptimal suitability for antigenic analysis of A(H1N1)pdm09 viruses, given the higher dominance of site Sb over site Sa found in humans^38^. In contrast, SING/16 HA1 displays a more complex hierarchy of antigenic dominance with site B playing a key role in the antigenic drift of KS/14, suggesting that ferret anti-SING/16 sera may be more effective for monitoring antigenic evolution of A(H3N2) viruses. In all, these findings provide valuable insights into assessment of ferret sera-based antigenicity data.

For the first time, we have demonstrated that ferret anti-HA1 serum Abs predominantly target classically defined antigenic sites of H1 and H3 HA using the Δ5-H1 and Δ5-H3 mutants. This finding is unexpected, given that the substitutions in both mutants account for only ~11% of HA1 residues (31 out of 282 HA1 residues for MI/45 Δ5-H1 and 30 out of 273 for SING/16 Δ5-H3). The proper folding of the two mutant rHA1s and the equivalent amounts of the WT and mutant rHA1s used in the assays have been validated, confirming that the substantial reduction in binding of the two mutants is due to the HA substitutions disrupting the HA1-sera interaction. This aligns with our previous findings, which show a strong correlation between HA1 binding and HI titers, a measure of neutralizing Abs targeting the HA1 antigenic regions in and around the RBS^20^.

Our binding results for MI/45 rHA1s are consistent with previous HI data showing that site Sa is the dominant antigenic site in the ferret Ab response to MI/45 virus infection^38^. These findings also align with earlier HI assay-based studies identifying a highly focused ferret Ab binding epitope encompassing residues 153, 154 and 155 of the HA from the A/California/7/2009(H1N1)pdm09 (CA/09) virus^20,35,45^. Amino acid sequence alignment of HAs from A(H1N1)pdm09 vaccine viruses (2010-2021) reveals a conserved KKGNS motif at positions 153-157, suggesting consistent dominant Ab responses against the H1 antigenic site Sa in these strains in ferrets. However, an N156K substitution has emerged in A(H1N1)pdm09 vaccine virus HAs from 2022 onward (Table 5). Further investigation is needed to determine whether this substitution affects the immunodominance of site Sa in ferrets. The substantial reduction in MI/45 Δ5-H1 binding compared to the limited changes in the binding of ΔSb, ΔCa1, ΔCa2 and ΔCb suggests the presence of serum Abs targeting epitopes that span multiple antigenic sites. Such Abs have been identified with epitopes encompassing residues in sites Sa, Sb and Ca2^46–48^.

**Table 5:** Aminoacid sequences of the 2010–2025 Northern Hemisphere A(H1N1)pdm09 vaccine virus HAs (143–160)

^a^NH (Northern Hemisphere).

^b^The aminoacid sequence of indicated virus HA from positions 143 to 160 with the KKGNS motif underlined and substitutions highlighted.

The antigenic dominance hierarchy of SING/16 HA1 suggests that ferret anti-SING/16 sera may effectively detect A(H3N2) virus variants with mutations in H3 antigenic sites B, C, or E. This is supported by our results demonstrating the dominant role of site B residues Y159 and F193 in the KS/14 antigenic drift. This is the first report providing the molecular basis for the KS/14 antigenic drift. Site B is known to be immunodominant in human Ab responses to current A(H3N2) vaccines, with single amino acid substitutions at seven positions including 159 and 193 that drive major antigenic changes in H3 HA^15,39,44,49^. Given the critical functional roles of residues at these two positions, HI-active Ab binding to Y159 and F193 in the HA-sera interaction is highly likely. Residue Y159 is essential for SING/16 HA1 binding to the egg-adapted sera but has minimal impact on the WT sera binding. This difference is likely due to de-glycosylation at residue N158 caused by the egg-adaptive T160K substitution, which exposes Y159 for dominant Ab binding. In contrast, F193 is positioned farther from N158, making its antigenicity potentially less affected by glycosylation changes at this position (Fig. 1B). Furthermore, our findings on antigenic drift induced by egg-grown A(H3N2) viruses align with the low vaccine effectiveness observed with egg-based A(H3N2) influenza vaccines from the 2016-2020 influenza seasons^22,24,41,42,50^. In contrast, similar effectiveness was found between the cell-based and egg-based vaccines from the 2022-2023 influenza season, consistent with our BLI binding results for DRW/6 rHA1s^51^. Comparative data for the effectiveness of 2021-2022 influenza vaccines are not available. All the egg-grown A(H3N2) viruses causing antigenic drift carry HA mutations that lead to HA de-glycosylation, including T160K in HK/4801, SING/16, and CBDA/20, as well as N246T in KS/14. This is consistent with previous observations that changes in HA glycosylation are strongly associated with major antigenic drift^12,22,52^.

One major limitation of this study is the lack of functional analysis to assess the contribution of the identified amino acid residues to neutralizing Ab binding. Future studies using HI and MN assays with reverse genetics-derived recombinant influenza viruses carrying mutations of these residues would help confirm their functional significance. Additionally, epitope mapping of human sera would provide further insight into the clinical relevance of these binding sites. Lastly, insect cells predominantly produce simple, high mannose or paucimannose N-glycans, whereas mammalian cells generate complex, branched N-glycans containing terminal galactose and/or sialic acids^53^. Consequently, the insect cell-derived rHA1 proteins used in this study may not fully recapitulate the glycosylation patterns of HAs expressed in mammalian cells, potentially leading to differences in HA antigenicity. Interestingly, a previous study analyzing the glycan-dependent immunogenicity of recombinant H5 HA in chickens and mice showed that insect cell-derived HA proteins carrying terminal mannose moieties induced lower HI titers compared to their mammalian cell-derived counterparts possessing complex glycans or single N-acetylglucosamine side chains^54^. However, recombinant HAs produced in mammalian cells present a set of issues as they can be expressed as sialylated species that multimerize and can affect studies, as shown previously by Wei et al.^55^. On the other hand, influenza viruses themselves have neuraminidase (NA) that ensures complex glycans on viral HAs terminate in galactose. Therefore, from a bulk perspective, the sizes of N-glycans on recombinant HAs generated in insect cells can be comparable to those of viral HAs produced in influenza virus-infected mammalian cells, potentially resulting in similar immunogenicity. This notion could be supported by the observation that the US Food and Drugs Administration (FDA)-approved Flublok vaccine, which is based on recombinant HA expressed in insect cells, has demonstrated vaccine effectiveness comparable to that of egg- and mammalian cell-derived influenza vaccines^56^. Future studies using rHA1s co-expressed with NA in mammalian cells may provide additional insights into the impact of glycan structures on HA antigenicity. Given the species-specific nature of immune responses to influenza virus infection and the influence of immune imprinting on antibody binding, our findings underscore the importance of incorporating human sera into antigenic analyses of influenza viruses.

## Methods

### Cloning and expression of rHA1 proteins

Codon optimized cDNAs encoding the wild type (WT) HA1 domains of A/Michigan/45/2015(H1N1)pdm09 (MI/45) HA (residues 31-312, H1 numbering) and A/Singapore/INFIMH-16-0019/2016(H3N2) (SING/16) HA (residues 41-313, H3 numbering) were synthesized (GenScript USA Inc., NJ) and sub-cloned into pIEx-4 vector (EMD Millipore, MA) using the In-Fusion HD cloning system (Clontech, CA). cDNAs encoding panels of antigenic site mutants of rHA1, each carrying multiple substitutions within the antigenic sites of H1 or H3 HA, were similarly synthesized and sub-cloned (Table 1). To generate these antigenic site mutants, the classically defined H1 antigenic sites of MI/45 were partially substituted with the corresponding sequences of the HAs from A/Vietnam/1203/2004(H5N1) and A/black headed gull/Sweden/1/1999(H13N6)^38^. Similarly, the classically defined H3 antigenic sites of SING/16 were partially replaced with the sequences of HA from A/Jiangxi-Donghu/346-1/2013(H10N8) with modifications^39^. HA1 constructs each carrying one amino acid substitution were generated from the WT pIEx-4-HA1 clones of MI/45 or SING/16 using the QuickChange Lightning Site-Directed Mutagenesis Kit (Stratagene, CA) (Table 2). Additionally, expression vectors of the WT and mutant HA1s of A/Kansas/14/2017(H3N2) (KS/14) were also similarly generated (Table 3). Lastly, cDNAs encoding the WT HA1s and corresponding mutants carrying egg-adaptive mutations of recent seasonal A(H3N2) vaccine viruses including A/Texas/50/2012 (TX/50), A/Switzerland/9715293/2013 (SWI/13), A/Hong Kong/4801/2014 (HK/4801), SING/16, KS/14, A/Cambodia/e0826360/2020 (CBDA/20), and A/Darwin/6/2021 (DRW/6), were similarly synthesized and subcloned (Table 4). All the rHA1 constructs were transiently transfected into Spodoptera frugiperda Sf9 cells (EMD Millipore, MA) using the Cellfectin II transfection reagent (Thermo Fisher Scientific, MA). All procedures were performed following protocols provided by the manufacturers. The transfected cells were grown in suspension on an orbital shaker at 27 °C for five days. All rHA1 proteins contained a signal sequence for secretion, a thrombin site at the C-terminus followed by a trimerizing sequence (foldon) from the bacteriophage T4 fibritin for generating functional trimers, and a His-Tag to aid detection. The expression levels of rHA1s secreted in the culture supernatant were quantified by chemiluminescent Western blot analysis using Penta-His antibody (Qiagen, CA) and a ChemiDoc MP imaging system (Bio-Rad, CA) following the manufacturer’s directions. The concentrations of mutant rHA1s were determined by densitometric analysis and normalized to the corresponding WT rHA1. Equivalent amounts of rHA1s used in the binding assay were validated by Western blot and applied in epitope mapping analysis without further purification.

### Cloning and expression of monoclonal antibody FluA-20

Amino acid sequences encoding the variable domains of heavy and light chains of monoclonal antibody FluA-20 (PDB ID: 6OBZ) were reverse translated. The resulting cDNAs were codon-optimized, synthesized (GenScript USA Inc.) and sub-cloned into vectors pFUSEss-CHIg-hG1 or pFUSE2ss-CLIg-hk (InvivoGen, CA). The expression vectors of the FluA-20 heavy and light chains were co-transfected into 293 T cells using lipofectamine 3000 (Thermo Fisher Scientific, MA) following instructions provided by the manufacturer. The transfected cells were transferred to a CO_2_ incubator and incubated in DMEM media containing 10% ultra-low IgG fetal calf serum (Thermo Fisher Scientific) at 37 °C for 3 days. The expressed IgG antibody was purified from the culture supernatant using a protein A column (Cytiva, MA) and a peristaltic pump (Bio-Rad, CA) following protocols provided by the manufacturers.

### f-AbBA-2

Epitope mapping of HA1 for ferret serum Abs was performed by the f-AbBA-2 using an Octet RED384 system (Sartorius, CA) as described previously^20^. Briefly, the transiently expressed rHA1 proteins of equivalent amount were coupled to anti-penta-His biosensors by incubating the tip of biosensors into the supernatant of recombinant proteins in kinetics buffer with the usage of a sidekick biosensor immobilization station (Sartorius, CA). Sera were diluted in kinetics buffer, and binding was analyzed by BLI on an Octet RED384 system. The assay steps were defined as the following: baseline for 120 seconds, association for 300 seconds, and dissociation for 120 seconds. Data were analyzed using the system software. Binding to the biosensor tip was measured as a wavelength shift (in nanometer) and end-point binding of the association step was used as a quantification of the binding avidity of serum Abs for recombinant proteins. In addition, the proper folding of expressed H1 or H3 rHA1s was confirmed by the BLI binding assay as described above using monoclonal antibodies (mAbs) IT-3A10 (Catalogue number MIA-H1-M015, eENZYME, MD) and FluA-20^40^, respectively.

### Ferret sera

Sera from ferrets infected with cell-grown or egg-grown viruses were provided by the Virology, Surveillance and Diagnosis Branch at the Centers for Disease Control and Prevention (CDC) or the International Reagent Resource (IRR) (Table 4). All sera were pretreated with receptor-destroying enzyme (RDE, Denka Seiken, Tokyo, Japan) following the manufacturer’s instructions and diluted to working stocks with phosphate-buffered saline (PBS), pH 7.2.

### Statistical analysis

Two-tailed paired t-tests were performed using GraphPad Prism 10.2.2 (GraphPad Software, CA). Significance levels are indicated with asterisks: ^*^p < 0.05, ^**^p < 0.01, ^***^p < 0.001, ^****^p < 0.0001, and ns (not significant).

## Supplementary information

Supplementary Information

## Data Availability

All data generated or analyzed in this study are included in this published article and its Supplementary Information. The raw data files used to generate figures are available upon request to the corresponding authors. Sequences of cell- and egg-version HAs used in this study are publicly available from GISAID with the following accession numbers: EPI662594 (MI/45-c), EPI685579 (MI/45-e), EPI377499 (TX/50-c), EPI731465 (TX/50-e), EPI530687 (SWI/13-c), EPI1868552 (SWI/13-e), EPI675798 (HK/4801-c), EPI1026711 (HK/4801-e), EPI1106235 (SING/16-c), EPI1151800 (SING/16-e), EPI1653968 (KS/14-c), EPI1261067 (KS/14-e), EPI1837753 (CBDA/20-c), EPI1883088 (CBDA/20-e), EPI1857216 (DRW/6-c), and EPI1925255 (DRW/6-e).
