# Supplementary material for: Immunodominant antibodies against hemagglutinin in ferrets following infection with 2018 to 2019 influenza vaccine strains
Source: NPJ Vaccines. 2025 Nov 21;10:253. doi: 10.1038/s41541-025-01282-y (PMC12669751; doi:10.1038/s41541-025-01282-y)
Supplement: Supplementary file 1 — Supplementary Information [file 41541_2025_1282_MOESM1_ESM.pdf]

Supplementary information for

Immunodominant antibodies against hemagglutinin in ferrets following infection with 2018 to 2019 influenza vaccine strains

Zhu Guo[1,✉], Thomas Rowe[1], Jessie Chang[1], Paul J. Carney[1], John Steel[1], and James Stevens[1,✉]

[1]Influenza Division, National Center for Immunization and Respiratory Diseases, Centers for Disease Control and Prevention, 1600 Clifton Road, Atlanta, GA 30329, USA. ✉Corresponding authors E-mail: bkj2@cdc.gov; fwb4@cdc.gov.

This PDF file includes:

Supplementary Figures 1 to 5

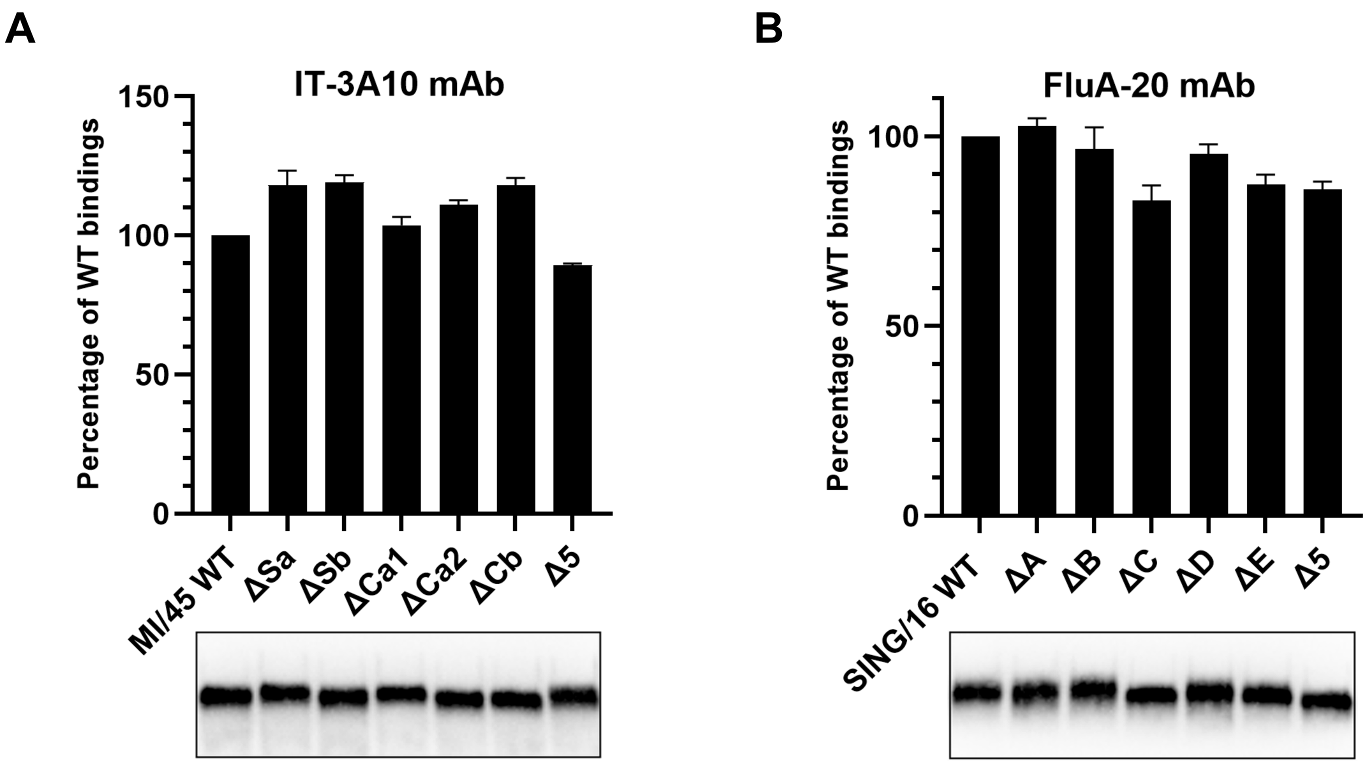

Supplementary Figure 1. Characterization of antigenic site mutants of MI/45 and SING/16 HA1. The proper folding of antigenic site mutants of MI/45 and SING/16 rHA1 was assessed using the f-AbBA-2 with the anti-MI/45 HA1 mAb, IT-3A10 **(A)** and the pan-H3 anti-HA1 mAb, FluA-20 **(B)**, respectively. Binding results for each rHA1 mutant were normalized against corresponding WT controls. Each bar represents the median and standard deviation from three independent experiments. Equivalent amounts of rHA1s used in the assay were confirmed by Western blot.

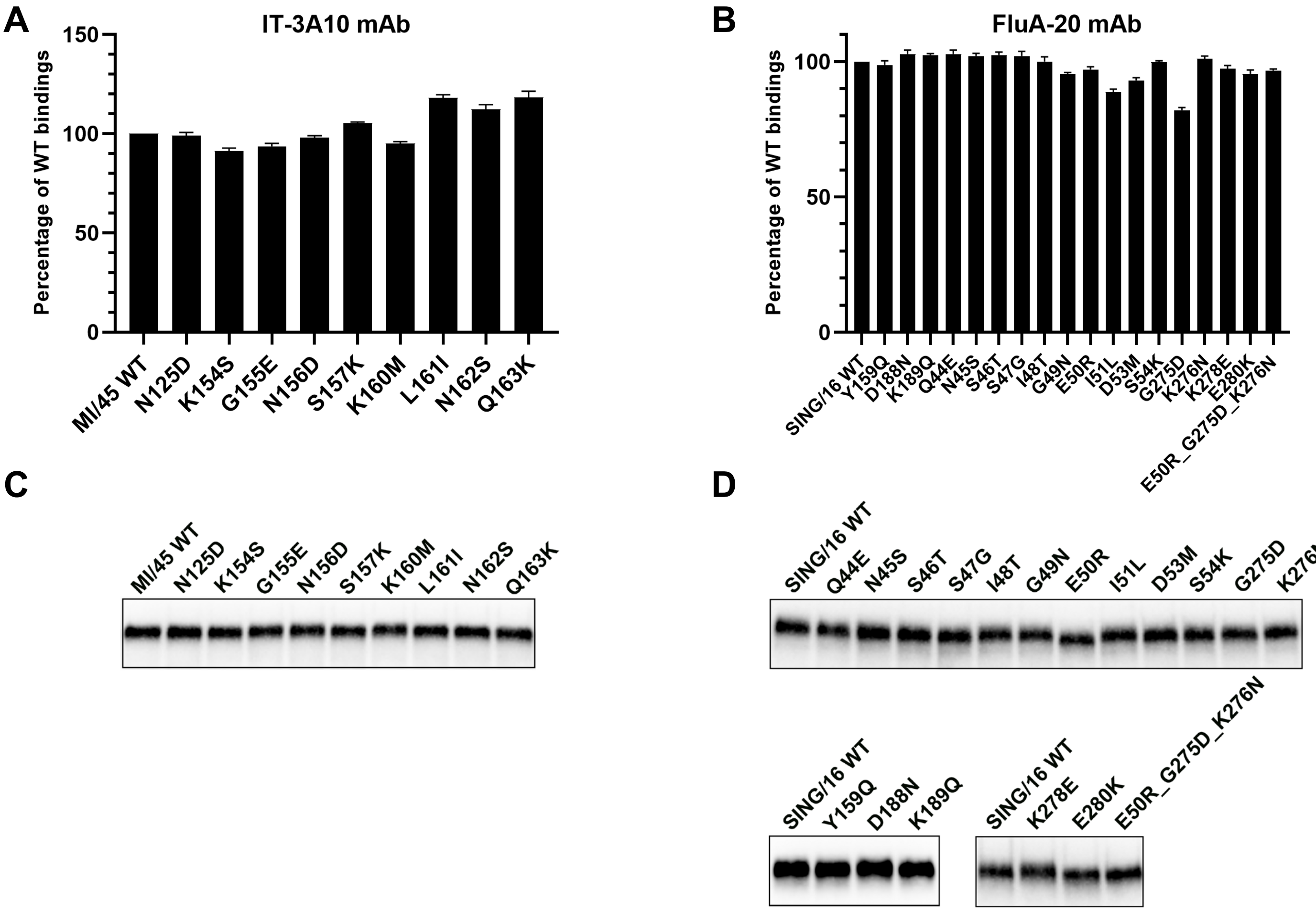

Supplementary Figure 2. Characterization of rHA1 mutants carrying single or multiple substitutions within dominant antigenic sites of MI/45 and SING/16. The proper folding of MI/45 and SING/16 rHA1 mutants was assessed using the f-AbBA-2 with the anti-MI/45 HA1 mAb, IT-3A10 **(A)** and the pan-H3 anti-HA1 mAb, FluA-20 **(B)**, respectively. Binding results for each rHA1 mutant were normalized against corresponding WT controls, with each bar representing the median and standard deviation from three independent experiments. Equivalent amounts of rHA1s used in the assay were confirmed by Western blot **(C & D)**.

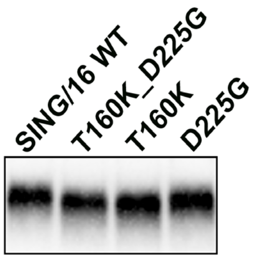

Supplementary Figure 3. Characterization of SING/16 rHA1 mutants carrying single or multiple egg-adaptive substitutions. Equivalent amounts of rHA1s used in the assay were confirmed by Western blot.

A

```
                 50         60         70         80         90        100        110        120
SING/16   ELVQNSSIGE ICDSPHQILD GENCTLIDAL LGDPQCDGFQ NKKWDLFVER SKAYSNCYPY DVPDYASLRS LVASSGTLEF
KS/14     ELVQNSSIGE ICDSPHQILD GENCTLIDAL LGDPQCDGFQ NKKWDLFVER NKAYSNCYPY DVPDYASLRS LVASSGTLEF

                130        140        150        160        170        180        190        200
SING/16   KNESFNWTGV TQNGTSSACI RGSSSSFFSR LNWLTHLNYT YPALNVTMPN KEQFDKLYIW GVHHPGTDKD QIFLYAQSSG
KS/14     NNESFNWAGV TQNGTSSSCI RGSKSSFFSR LNWLTHLNSK YPALNVTMPN NEQFDKLYIW GVHHPGTDKD QISLYAQSSG

                210        220        230        240        250        260        270        280
SING/16   RITVSTKRSQ QAVIPNIGSR PRIRDIPSRI SIYWTIVKPG DILLINSTGN LIAPRGYFKI RSGKSSIMRS DAPIGKCKSE
KS/14     RITVSTKRSQ QAVIPNIGSR PRIRDIPSRI SIYWTIVKPG DILLINSTGN LIAPRGYFKI RSGKSSIMRS DAPIGKCKSE

                290        300
SING/16   CITPNGSIPN DKPFQNVNRI TYGACPRY
KS/14     CITPNGSIPN DKPFQNVNRI TYGACPRY
```

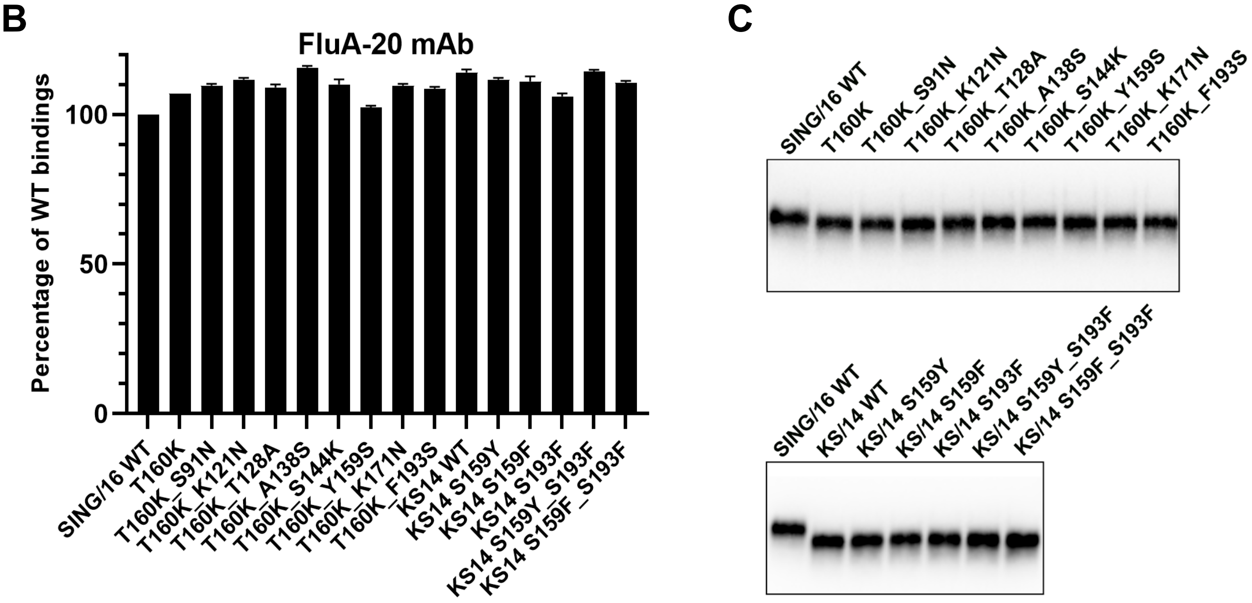

Supplementary Figure 4. Characterization of SING/16 and KS/14 rHA1 mutants used in SING/16 HA1 epitope mapping. (A) Amino acid sequences of SING/16 and KS/14 HA1 were aligned with substitutions highlighted. **(B)** The proper folding of expressed rHA1 mutants from SING/16 and KS/14 was assessed using the f-AbBA-2 with the pan-H3 anti-HA1 mAb, FluA-20. Binding results for each rHA1 mutant were normalized against SING/16 WT controls, with each bar representing the median and standard deviation from three independent experiments. **(C)** Equivalent amounts of rHA1s used in the assay were confirmed by Western blot.

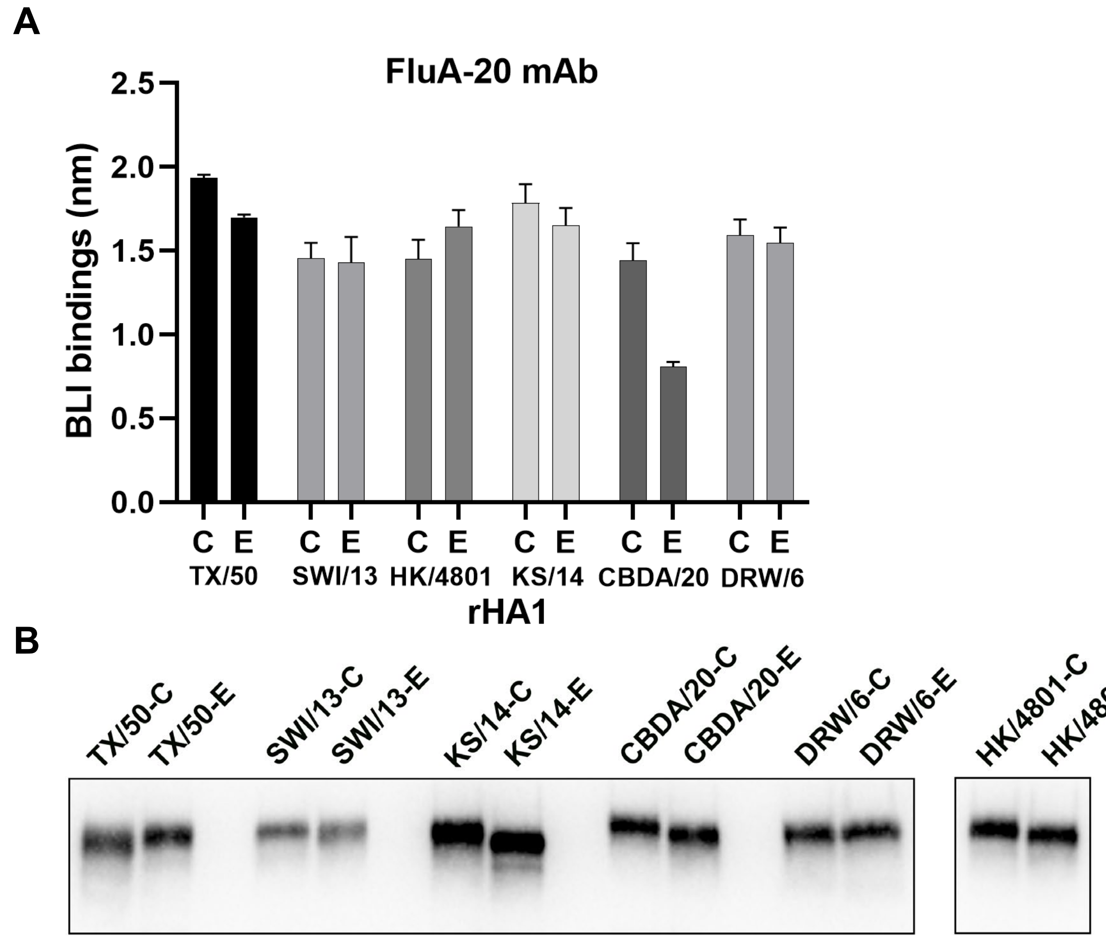

Supplementary Figure 5. Characterization of rHA1s of A(H3N2) vaccine viruses from recent flu seasons. (A) The proper folding of expressed rHA1s from cell-grown or egg-grown A(H3N2) vaccine viruses was assessed using the f-AbBA-2 with the pan-H3 anti-HA1 mAb, FluA-20. Each bar represents the median BLI binding with standard deviation from three independent experiments. "C" denotes cell-version rHA1 from cell-grown virus, while E denotes egg-version rHA1 from egg-grown virus. **(B)** Equivalent amounts of each rHA1 pair used in the assay were confirmed by Western blot.
